# The Mathematical Foundations of 3D Compton Scatter Emission Imaging

**DOI:** 10.1155/2007/92780

**Published:** 2007-04-30

**Authors:** T. T. Truong, M. K. Nguyen, H. Zaidi

**Affiliations:** ^1^Laboratoire de Physique Théorique et Modélisation, CNRS UMR 8089, Université de Cergy-Pontoise, 2 Avenue Adolphe Chauvin, 95302 Cergy-Pontoise, France; ^2^Laboratoire Equipes de Traitement des Images et du Signal, CNRS UMR 8051, Ecole Nationale Supérieure de l'Electronique et de ses Applications, Université de Cergy-Pontoise, 6 Avenue du Ponceau, 95014 Cergy-Pontoise, France; ^3^Division of Nuclear Medicine, Geneva University Hospital, 1211 Geneva 4, Switzerland

## Abstract

The mathematical principles of tomographic imaging using detected (unscattered) X- or gamma-rays are based on the two-dimensional Radon transform and many of its variants. In this paper, we show that two new generalizations, called conical Radon transforms, are related to three-dimensional imaging processes based on detected Compton scattered radiation. The first class of conical Radon transform has been introduced recently to support imaging principles of collimated detector systems. The second class is new and is closely related to the Compton camera imaging principles and invertible under special conditions. As they are poised to play a major role in future designs of biomedical imaging systems, we present an account of their most important properties which may be relevant for active researchers in the field.

## 1. INTRODUCTION

During the last fifty years, progress in imaging systems using penetrating radiation for biomedical purposes has brought about new topics in mathematics and fueled intense research activities with far reaching results. The mathematics of imaging science has evolved to a full fledged discipline [1]. Transmission computer assisted tomography (CAT-scanning) is based on an integral transform in two dimensions discovered in the sixties by Cormack [2, 3, 4], who did not realize that J. Radon had already introduced and studied it in his seminal paper [5] in 1917. Subsequently, in an effort to reconstruct directly in three dimensions an object without having to assemble its two-dimensional sections, one is led to consider the so-called X-ray transform or cone beam transform. This transform is an off-spring of the Radon transform and maps a function in ℝ^3^ to its (straight) line integrals in ℝ^3^. One way to reconstruct an object is to convert the line data into planar data in ℝ^3^ using the Grangeat technique [6]. Then application of the inversion formula of the three-dimensional Radon transform [7] yields the answer. A further generalization of the Radon transform in emission imaging, called the attenuated X-ray transform, accounts for the radiation loss in the traversed medium. The problem of its inversion has been a mathematical challenge for decades and has been solved only in 2001 [8], thanks to complex analysis methods applied to the stationary photon transport problem [9]. The success of this branch of mathematics, coined by Gelfand as integral geometry, goes far beyond the imaging science scope as it has brought significant progress in group representation theory, partial differential equations, boundary value problems, and so forth [7].

The standard Radon transform and its variants are related to a process of data collection in an interaction free propagation of radiation through matter, possibly affected by attenuation. Thus, radiation energy is not altered from emission to detection. However, a sizable part of traveling radiation does suffer an interaction with matter through Compton scattering [10]. Generally as the data quality is lowered, Compton scattering effects have been treated as noise and must be eliminated. So far, in most image processing methods, the aim is to deal away with it, for example, by filtering or by geometric rejection using an antiscatter grid of miniature lead septa. In conventional projection imaging, most of the radiation have been scattered in the patient body, the scatter-to-primary ratio can be as high as 10 [10]. But throwing out all the scattered radiation may not be a smart move since this also means a loss of sensitivity and certainly a loss of valuable information.

This is why we have recently advocated the use of scattered radiation to improve quality in image processing [11] as well as to construct new imaging principles [12]. This proposal generalizes the concept of a Compton camera, proposed by several workers some thirty years ago [13–15], in which the idea of electronic collimation is implemented. When radiation is detected at a lower energy than the originally emitted energy, there must be at least one Compton scattering occurring along its propagation path. But the vast majority of scattered radiation is due to mainly single scattering events [16]. To an emerging ray detected at a definite energy there corresponds an ensemble of incoming rays distributed on a circular cone of definite opening angle. The measured data consisting of collected emerging radiation at some detection point is viewed as the integral contribution of a source function on specific circular cones; it may be also called a conical projection of the source. The name of conical Radon transform is attributed to such integral transform and is added to the list of already known Radon transforms on geometric manifolds in ℝ^3^ : paraboloids [17], spheres [18], special surfaces [19], second-order surfaces [20], and so forth. Other proposals for use of scattered radiation in X-ray imaging which do not rely on conical projections have been made independently by [21].

Thus, the main topic addressed here is the mathematical framework defining the working principles of a new imaging technique. To keep the discussion transparent, we have adopted an ideal working assumption whereby attenuation is not taken into account. A well-known difficulty is that the nonuniform attenuation along the radiation path leads to tremendous mathematical complication. For a given projection, attenuation correction factors in SPECT rarely exceed 10 in virtually all clinical imaging situations [22]. An exact solution is beyond the scope of the present study. It should be pointed out that, in the case of the attenuated X-ray transform in emission imaging, a comprehensive analytic solution has been attained only recently [9]. Therefore, in practical situations standard corrections for photon attenuation should be envisaged.

In conventional emission imaging, the data collected for object reconstruction is formed by linear projections (cone beam projections) of unscattered radiation filtered by an energy acquisition window which has already discarded from 70% to 80% of the incident photon flux. Moreover, among the retained some have suffered scattering in the collimator and hence must be discarded. It has been reported that collimator scatter increases as the energy of the photopeak of interest increases from a low of 1.9% for Tc-99 m (141 keV) to a high of 29.4% for I-131 (364 keV) with the usual high-energy collimator. The penetration percentage also goes up with energy. Therefore, correction for photons that penetrate through, or scatter in, collimator septa is hardly important at all for Tc-99 m tracers [23].

In the proposed imaging technique, data for reconstruction are provided by the so-called conical projections, which gather radiation from point sources lying on large surfaces inside the emitting object. Although contamination of the scatter component by multiple scatter events may be as high as 30% of the total scatter according to Monte Carlo investigations [16, 24], we believe that the signal of single scattered radiation is largely sufficient to make the new imaging principles work, in particular when advanced semiconductor-based detectors with better energy and spatial resolution and sensitivity will become available. These interesting issues will be explored in future work.

In this paper, we present a unified treatment of conical Radon transforms relevant in emission imaging by scattered radiation. In fact, we will be concerned with two classes of conical Radon transforms originating from image formation by Compton scattered radiation on a gamma camera with and without collimator. The first class of conical Radon transform uses circular cone sheets with fixed axis direction (*𝒞*_1_-cones) whereas the second class deals with circular cone sheets with axis swinging around a point (*𝒞*_2_-cones). Note that if this point goes to infinity in a given spatial direction, then the *𝒞*_2_-cone goes over the *𝒞*_1_-cone. In each case, we will start by showing how the image formation process leads to an integral transform and how this transform is related to the conical Radon transforms. Each conical transform will be introduced and its relevant properties for imaging purposes, in particular their invertibility, discussed. Conclusions and perspectives are given in the last section.

## 2. NOTATIONS

Let *f* be a real nonnegative integrable, smooth, with compact support function on ℝ^3^. The same function in cylindrical (spherical) coordinates is noted f(f).

The definitions of various transforms of *f*(*x*,*y*,*z*) are as follows:

- ${\hat{f}}_{i}$: Conical *𝒞*_i_-Radon transform of *f*, *i* = 1, 2;
- **F**_j_: *j*-dimensional Fourier transform of *f*, *j* = 1, 2, 3.

Special functions [25] are as follows:

- *Y* (*x*): Heaviside unit step function;
- *j_l_*(*kr*): spherical Bessel function of order *l* and variable (*kr*);
- *P_l_*(cos *θ*): Legendre polynomial of order *l* and of variable cos*θ*;
- *Y_l,m_*(Ω_*k*_): spherical harmonic (*l,m*) with argument Ω_*k*_, solid angle in the direction of the unit vector **k**.

## 3. WORK SETTING

**Figure 1:**
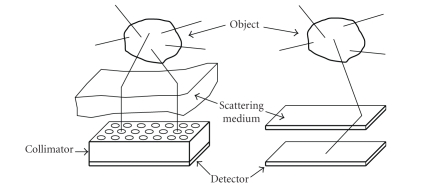
Two imaging modalities using scattered radiation with and without collimation.

In this article, we consider the emission imaging problem, that is, the problem of reconstructing in ℝ^3^ a gamma-ray radiating object from its Compton scattered radiation data. This object is described by its activity volume density function *f*. Detection of scattered radiation is performed by a gamma camera in two instances: with or without collimator. The recorded data consists of the coordinates of the detection site, the surface flux density of photons at this site (pixel), and the value of their energy (list mode). Between the radiating object and the detector stands a scattering medium: it may be a volume or a layer as illustrated by Figure 1. Note that the object itself may also be a scattering medium, and for photon energies above 25 keV, over 50% of the interactions in biological tissues are scatterings [26]. Higher-order scattering events (of much lower probability of occurrence) will be the object in future studies.

### 3.1. Compton scattering

As Compton scattering plays a key role, we will recall some of its properties. The Compton effect discovered in 1923 [27] had served to confirm the particle (photon) nature of radiation, as proposed by A Einstein. Thus, energetic radiation under the form of X- or gamma-rays behave like particles and scatter with electrically charged particles in matter. In biomedical domains, X- or gamma-photons scatter electrons in the biological media they traverse. This scattering process has cylindrical symmetry around the direction of the incoming photon and the energy of the outgoing photon is given by the Compton formula [28]

(1) $$E=E_{0}\frac{1}{1+\varepsilon (1-\cos\omega )},$$

where *ω* is the scattering angle as measured from the incident photon direction, *E*_0_, the photon initial energy, *ε* = *E*_0_/*m* *c* ^2^, and *m* *c* ^2^ the rest energy of the electron. Equation (1) shows that single-scattered photons have a continuous energy spectrum in the range *E*_0_/(1 + 2*ε*) ≤ *E* ≤ *E*_0_.

Thus an emerging photon with an energy *E* in a direction of unit vector **n** may originate from an incoming photon of energy *E*_0_ emitted from a site on a sheet of a circular cone of axis direction **n** and an opening angle *ω*.

### 3.2. Compton differential cross-section

**Figure 2:**
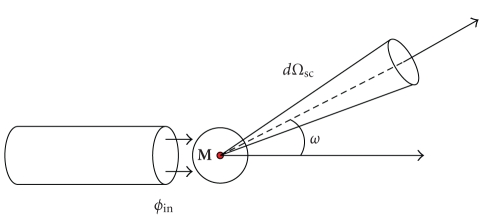
Compton scattering differential cross-section.

At a scattering site **M**, the number of particles *d* ^2^ *N*_sc_ scattered in a solid angle *d*Ω_sc_ along a direction making an angle *ω* with the incident direction follows from the definition of the differential scattering cross-section (see Figure 2)

(2) $$\frac{d\sigma_{C}}{d\Omega },$$

when the following quantities are given:

- *ϕ*_in_, the incident photon flux density,
- *n_e_*(**M**)*d* **M**, the number of scatterers (electrons) around the scattering site **M** with volume *d* **M**.

We have

(3) $$d^{2}N_{\text{sc}}=\phi_{in}n_{e}(M)dM(\frac{d\sigma_{C}}{d\Omega })d\Omega_{\text{sc}}.$$

The Compton differential cross-section has been computed in 1929 by Klein and Nishina [29]. It appears as the product of the area of a disk of radius *r_e_*, the classical radius of the electron, and a probability factor *P*(*ω*), that is,

(4) $$(\frac{d\sigma_{C}}{d\Omega })=(\pi r_{e}^{2})P(\omega ),$$

where *r_e_* = 2.82 × 10 ^− 15^ *m* and

(5) $$P(\omega )\text{​}=\text{​}\frac{1}{2\pi }\frac{1}{{\left[1+\varepsilon (1\text{​}-\text{​}\cos\;\omega )\right]}^{2}}(1\text{​}+\text{​}\cos^{2}\omega +\frac{\left(1\text{​}-\text{​}\cos^{2}\;\omega \right)}{1\text{​}+\text{​}\varepsilon (1\text{​}-\text{​}\cos\;\omega )}).$$

Thus, we see that, for a given incident energy, the angular distribution of scattered photons is no longer isotropic [28].

Hence, the final form of this number of scattered photons in the direction given by the angle *ω* is

(6) $$d^{2}N_{\text{sc}}=\phi_{\text{in}}n_{e}(M)dMr_{e}^{2}P(\omega )d\Omega_{\text{sc}},$$

this number is basic to the image formation by scattered radiation.

### 3.3. Conical projections

In standard computer assisted tomography (CAT), the data is gathered under the form of line projections or integrals of a function (attenuation or activity) along straight lines. Here following the scattering mechanism, the data would appear as conical projections or integrals of a function *f* on a circular cone sheets. The integration is carried out with the Lebesgue measure of the cone in a chosen coordinate system. The result is a function of

- the coordinates of the cone vertex,
- the parameters of the unit vector of the cone axis,
- the opening angle of the cone, that is, *ω* the scattering angle.

**Figure 3:**
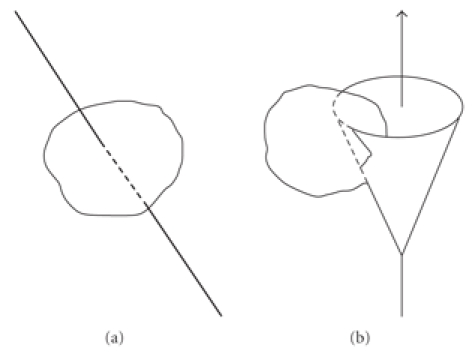
Illustration of linear and conical projections.

Figure 3 displays the representations of line projection and conical projection. In the text we will have two types of conical projections corresponding to the two cases of image formation mentioned earlier. The question is now how to use the conical projections to reconstruct the source function *f*. We will treat the two cases separately in the coming sections.

## 4. THE*𝒞*_1_-CONICAL RADON TRANSFORM

In this section, we consider the possibility of imaging a three-dimensional object by collecting data on its scattered radiation on a gamma camera equipped with a collimator and show how the *𝒞*_1_-conical Radon transform arises. Figure 1 shows the experimental arrangement with the location of the radiating object, the scattering medium, and the collimated gamma camera.

### 4.1. Image formation in gamma imaging by scattered radiation

by scattered radiation

To concentrate on the scattered imaging principle, we make some simplifying assumptions [12, 30]:

1. absence of attenuation for the propagating radiation,
2. constant density of the electrons *n*_e_ in the scattering medium,
3. isotropic emission from original radioisotopes in object.

To compute the photon flux density at a detection site (pixel) **D** on the collimated camera we start from (6). Radiation is emitted at point source **S**, will propagate to scattering site **M** and reach detection site **D**.

The incoming photon flux density *ϕ*_in_ on scattering site **M** is now computed from the emission data from point source. Let *f*(**S**)*d* **S** be the number of gamma photons emitted per unit time by a volume *d* **S** in the object around site **S**. The emission being isotropic, the number of photons emitted in the direction $\vec{SM}$ in a solid angle *d*Ω*_S_* is

(7) $$\frac{f(S)dS}{4\pi }d\Omega_{S}.$$

Therefore, the incoming photon flux density at scattering site **M** is

(8) $$\frac{f(S)dS}{4\pi }\frac{1}{SM^{2}}=\phi_{\text{in}},$$

where $SM=|\vec{SM}|$.

Next, the number of scatterers around site **M** in a volume *d* **M** is *n_e_* *d* **M**. The net number of photons emerging from the scattering is

(9) $$\frac{f(S)dS}{4\pi }\frac{1}{SM^{2}}n_{e}\;dM\pi r_{e}^{2}P(\omega )d\Omega_{M},$$

this means that the detected photon flux density at site **M** is

(10) $$\frac{f(S)dS}{4\pi }\frac{1}{SM^{2}}n_{e}\;dM\pi r_{e}^{2}P(\omega )\frac{1}{MD^{2}}.$$

Now, all the contributing point sources **S**, for given scattering center **M**, lie on a circular cone sheet of axis identified with $\vec{MD}$ and opening angle *ω*, thus we must integrate with the measure *δ*(cone)*d* **S** first. Next, we must take into account all the scattering sites in the scattering medium situated on the line parallel to the collimator axis at site **M**. Hence, we must perform a second integration with the measure *δ*(line)*d* **M**. To sum up the detected photon flux density at **D** is

(11) $$\begin{array}{ll} g(D,\omega ) & =\iint \delta (cone)\frac{f(S)dS}{4\pi }\frac{1}{SM^{2}}n_{e}\;dM\pi r_{e}^{2}P(\omega )\\ & \;\times \delta (line)\frac{1}{MD^{2}}. \end{array}$$

**Figure 4:**
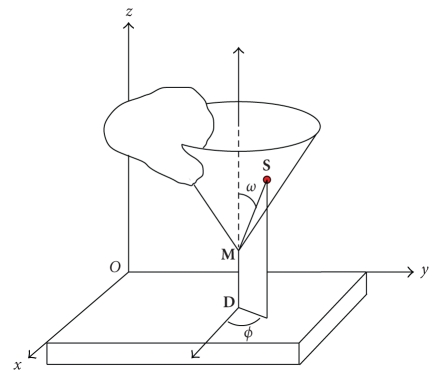
*𝒞*_1_-cone and *𝒞*_1_-conical Radon transform definition.

In the cylindrical coordinate system of Figure 4, the integration measure on the cone is *r* sin *ω dr dϕ* and the measure along the line is simply *dz_M_*, (11) becomes

(12) $$\begin{array}{ll} g(D,\omega )= & \pi r_{e}^{2}P(\omega )\frac{n_{e}}{4\pi }\iint r\;\sin\;\omega drd\phi \\ & \times f(x_{D}+r\;\sin\;\omega \;\cos\;\phi ,y_{D}\\ & \;\;\;\;+r\;\sin\;\omega \;\sin\;\phi ,z_{M}+r\;\cos\;\omega )\frac{dz_{M}}{z_{M}^{2}}. \end{array}$$

**Remark 1**

In practice as *f* and *n_e_* are volume densities, to keep the physical dimensions right, we should think of the cone sheet as having a small thickness *e* and the line on which **M** moves as having a tiny section *s*. These are constants and do not affect the mathematical reasoning, they will be dropped for the sake of expression simplicity, but should always be kept in mind.

Clearly this imaging equation is a compounded integral transform. Assuming that integration interchange is valid, if we define the first integral transform as

(13) $$\begin{array}{cc} h(x_{D}+r\;\sin\;\omega \;\cos\;\phi ,y_{D}+r\;\sin\;\omega \;\sin\;\phi ,r\;\cos\;\omega ) & \\ =\int \frac{dz_{M}}{z_{M}^{2}}f(x_{D}+r\;\sin\;\omega \;\cos\;\phi ,y_{D} & \\ \;\;\;\;+r\;\sin\;\omega \;\sin\;\phi ,z_{M}+r\;\cos\;\omega ), & \end{array}$$

we see that the imaging equation (12) is just a conical projection of the function *h* on the *𝒞*_1_-cones with vertex on the plane *xOy*.

Let us define an interaction factor which also includes *e* and *s*:

(14) $$K(\omega )=\pi r_{e}^{2}P(\omega )\frac{n_{e}}{4\pi }es,$$

then (12) reads

(15) $$\begin{array}{cc} \frac{g(D,\omega )}{K(\omega )}=\iint r\;\sin\;\omega drd\phi h( & x_{D}+r\;\sin\;\omega \;\cos\;\phi ,y_{D}\\ & +r\;\sin\;\omega \sin\phi ,r\;\cos\;\omega ), \end{array}$$

or in terms of our notations (see Section 2)

(16) $$\frac{g(D,\omega )}{K(\omega )}={\hat{h}}_{1}(x_{D},y_{D},\omega ).$$

### 4.2. Properties of the *𝒞*_1_-conical transform

The *𝒞*_1_-conical Radon transform has been discussed in [31], where some of its properties have been studied. However, the problem of inversion will be handled here with a new approach. We will not go through the method of decomposition of functions in circular components but will show that there exists a variant of the *central slice theorem* [28] which provides the grounds to invert the transform as it is done in the standard Radon transform [28].

First, we observe that by definition in the chosen cylindrical coordinate system the *𝒞*_1_-conical Radon transform of *f*, the cones having vertex on the *xOy* plane, is

(17) f^1(xD,yD,ω)=∬r sin⁡ ω dr dϕf(xD+r sin⁡ ω cos⁡ ϕ,yD+r sin⁡ ω sin⁡ ϕ,r cos⁡ ω).

This can be rewritten under the form of a Fredholm integral equation of the first kind with a delta function kernel concentrated on the sheet of a circular cone [31]

(18) f^1(xD,yD,ω) =∬dx dy dzK1(xD,yD,ω|x,y,z)f(x,y,z),

with

(19) $$\begin{array}{ll} K_{1}(x_{D},y_{D},\omega |x,y,z) & \\ \;=\delta (\cos\;\omega \sqrt{{\left(x-x_{D}\right)}^{2}+{\left(y-y_{D}\right)}^{2}}-z\;\sin\;\omega ). & \end{array}$$

We use now the Fourier representation of the delta function

(20) $$\begin{array}{ll} \delta (\cos\;\omega \sqrt{{\left(x-x_{D}\right)}^{2}+{\left(y-y_{D}\right)}^{2}}-z\;\;\sin\;\omega ) & \\ \;=\int_{-\infty }^{\infty }d\nu \;\exp\;2i\pi \nu (\cos\;\omega \sqrt{{\left(x-x_{D}\right)}^{2}+{\left(y-y_{D}\right)}^{2}} & -z\;\;\sin\;\omega ), \end{array}$$

and the two-dimensional Fourier transform of *f*(*x, y, z*,

(21) $$f(x,y,z)=\int dpdqe^{2i\pi (px+qy)}F_{2}(p,q,z).$$

We can perform the integration over *z*, which restores a three-dimensional Fourier transform *F*_3_(*p*, *q*, *r*) of *f* and yields a new form of $\hat{f}$ (*x_S_*, *y_S_*, *ω*),

(22) f^(xS,yS,ω)=∫dx dy∫−∞∞dν exp⁡ 2iπν(cos⁡ ω(x−xS)2+(y−yS)2)×∫dp dqe2iπ(px+qy)F3(p,q,ν sin⁡ ω).

Let ${\hat{F}}_{2}$ (*p*, *q*, *ω*) be Fourier component with respect to the coordinates *x_S_* and *y_S_* of $\hat{f}$ (*x_S_*, *y_S_*, *ω*), then

(23) F^2(p,q,ω)=∫−∞∞dνF3(p,q,ν sin⁡ ω)∬dx dy exp⁡2iπ×[ν cos⁡ ω(x−xS)2+(y−yS)2+(p(x−xS)+q(y−yS))].

The last integral of (23) can be computed using polar coordinates in (*x*, *y*) and (*p*,*q*) spaces, that is, (*x* − *x_S_*) = *ρ* cos *β* (*y* − *y_S_*) = *ρ* sin *β*, *dx* *dy* = *ρ* *d* *ρ* *d* *β*, and *p* = *k* cos *α*, *p* = *k* sin *α*, *k* *d* *k* *d* *α*. Therefore,

(24) $$\begin{array}{c} \iint dx\;dy\;\exp\;2i\pi \left[\nu \;\cos\omega \sqrt{{\left(x-x_{S}\right)}^{2}+{\left(y-y_{S}\right)}^{2}}+\left(p(x-x_{S})+q\left(y-y_{S}\right)\right)\right]\\ =\iint \rho \;d\rho \;d\beta e^{2i\pi [\nu \rho \;\cos\omega +k\rho \;\cos(\beta -\alpha )]}. \end{array}$$

Integration on *β* yields a Bessel function of order zero in the last integral of (24):

(25) $$\begin{array}{l} \iint \rho \;d\rho \;d\beta e^{2i\pi [\nu \rho \;\cos\;\omega +k\rho \;\cos\;(\beta -\alpha )]}\\ \;=\int_{0}^{\infty }\rho \;d\rho e^{2i\pi \rho \nu \;\cos\;\omega }2\pi J_{0}(2\pi k\rho ). \end{array}$$

Thus, in two-dimensional Fourier space, the *𝒞*_1_-conical Radon transform appears as

(26) F^2(p,q,ω)=∫−∞∞dν∫0∞ρ dρ2πJ0(2πρp2+q2)×F3(p,q,ν sin⁡ ω)e2iπνρ cos⁡ ω.

Now, we perform the integration on *ν* which brings back the three-dimensional Fourier transform of *f*,

(27) $$\begin{array}{l} \int_{-\infty }^{\infty }d\nu F_{3}(p,q,\nu \;\sin\;\omega )e^{2i\pi \nu \rho \;\cos\;\omega }=\frac{1}{\sin\;\omega }F_{2}(p,q,\rho \;\cot\;\omega ). \end{array}$$

This step reduces the *𝒞*_1_-conical Radon transform to a Hankel of order zero:

(28) sin⁡ωF^2(p,q,ω)=∫0∞ρdρ2πJ0(2πρp2+q2)F2(p,q,ρ cot⁡ ω).

To perform the inversion of this Hankel transform, we should switch to an appropriate variable, that is, *ζ* = *ρ* cot *ω*. But care must be exercised as far as the range of *ω* is concerned. We will distinguish two cases.

- 0 < *ω* < *π*/2, then *ζ* > 0 as well as tan *ω* > 0 and (28) can be rewritten as
  (29) sin⁡ ω cot⁡ 2ωF^2(p,q,ω<π2) =∫0∞ζdζ2πJ0(2πζ tan⁡ ωp2+q2)F2(p,q,ζ).
- *π*/2 < *ω* < *π*, then *ζ* < 0 as well as tan *ω* < 0 and (28) can be rewritten as
  (30) sin⁡ ω cot⁡2 ωF^2(p,q,ω>π2) =∫0∞ζdζ2πJ0(2πζ tan⁡ ωp2+q2)F2(p,q,−ζ).
  Application of the Hankel identity [25]
  (31) $$\frac{1}{k}\delta (k-k^{\prime })=\int_{0}^{\infty }rdr2\pi J_{l}(2\pi kr)2\pi J_{l}(2\pi k^{\prime }r)$$
  leads to the inversion of the Hankel transforms and yields the three-dimensional Fourier components of the object *f*. As the dual variable to *ζ* is $\tau \sqrt{p^{2}+q^{2}}$, with *τ* = tan *ω*, we get
  (32) F2(p,q,ζ)=(p2+q2)∫0∞τdτ2πJ0(2πζ tan⁡ ωp2+q2) ×1|τ|1+τ2(​Y(π2−ω​)F^2(​p,q,ω<π2)    +​Y(​ω​−​π2)F^2(p,q,ω>π2)​).

Hence, *f* can be recovered by inverse three-dimensional Fourier transform.

**Remark 2**

In order to reconstruct the object by inverting the compound integral transform given by (12), we need to invert (13). This is quite easy since it can be viewed as a convolution in the variable *z_D_* between the two functions 1/*z*_D_^2^ and *f*(… , … ,*z_D_* + ***r*** cos *ω*). As this operation is not important to the topic of this paper, we refer the reader to [12, [Bibr B30]].

## 5. THE *𝒞*_2_-CONICAL RADON TRANSFORM

### 5.1. Compton camera

That Compton effect which has been proposed as mechanism for imaging is known ever since the fifties. However, there are many ways to do the experimental setups. Most proposals are systems with collimated point source and point-like detector, see, for example, [21]. In fact the Compton effect is used to probe the electron density of matter and applied often to nondestructive material control. Here we are interested in data collected by a gamma camera. In the previous section we have examined the case of a gamma camera with a lead collimator which has the disadvantage of rejecting many of the scattered photons. So to improve drastically detection sensitivity, the idea of a Compton camera has been proposed as early as 1974 by many workers [13–15, 32].

**Figure 5:**
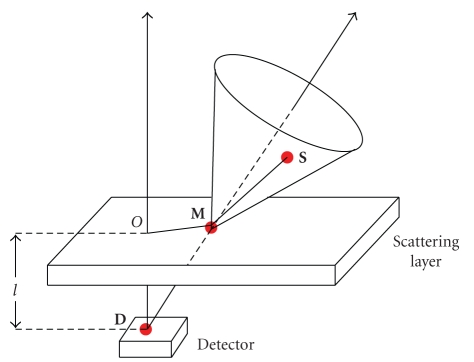
Principle of a Compton camera.

The concept of a Compton camera is analogous to the scheme of Section 4 except that the scattering medium is now a thin scattering layer parallel to the face of a gamma camera without collimator. The data consists of *𝒞*_2_-conical projections, the cone sheet axis converging to the detection site **D**; see Figure 5. Note that when **D** → ∞ in a given direction we recover the *𝒞*_1_ cones.

Following the image formation process in a Compton camera, we will show how a new conical Radon transform, the *𝒞*_2_-Radon transform, comes up and sketch a proof of its invertibility under specific conditions. The true conical Radon transform of a Compton camera is not yet an analytical inversion formula.

### 5.2. Image formation by scattered radiation in Compton camera

The radiating object stands above the first scattering layer. Its primary rays hit the scattering layer and will be absorbed by the planar camera (see Figure 5). If only photons of energy *E* below the energy *E*_0_ of the primary photons are recorded, then each detection site collects all possible conical projections coming from all directions in half space, delimited by the photon absorbing detector. This is the principle of electronic collimation which has been designed to improve sensitivity of gamma cameras.

To reconstruct an object described by a source function *f*(*x*, *y*, *z*), we need a set of data consisting of conical projections depending also on three variables. Ideally one could select one detection site **D**, and consider all the projections along circular cones of opening angle *ω* and axis swinging around **D** but with cone vertex constrained to be on a plane. With these conditions a conical projection will depend on three parameters: the scattering angle *ω* and the two coordinates of the cone vertex on the scattering plane. Thus, we obtain a mapping of *f* onto a function of three variables. The inverse mapping, when explicitly worked out, would yield a correct imaging procedure by a Compton camera.

Following the assumptions of Section 4.1, the photon flux density at detection site **D** is evaluated in the same manner as for the case with collimator:

(33) $$\begin{array}{cc} g(D,\omega )= & \iint e\delta (cone)\frac{f(S)dS}{4\pi }\frac{1}{SM^{2}}\\ & \;\times n_{e}dM\pi r_{e}^{2}P(\omega )s\delta (line)\frac{1}{MD^{2}}. \end{array}$$

Now in the chosen coordinate system (see Figure 5), it has the expression

(34) $$\begin{array}{cc} g(D,\omega )= & K(\omega )\iint \delta (cone)f(S)dS\frac{1}{SM^{2}}\\ & \;\times dx_{M}dy_{M}\frac{1}{l^{2}+x_{M}^{2}+y_{M}^{2}}, \end{array}$$

*l* being the distance *OD*, the explicit integration on the cone sheet *δ*(cone)*d* ***S*** will be given later in Section 5.3 since it is related to the *𝒞*_2_-conical Radon transform we will be examining.

Up to now there exists only a few attempts to exactly solve this inversion problem. Cree and Bones [33] were the first to consider conical projections on a Compton camera which still has a collimator. Later on Basko  [34] as well as Parra [35] have designed inversion techniques based on properties of spherical harmonics as they consider conical projections as made up of cone beam projections, but have not touch really upon the problem of converting cone beam data into conical projection data, a problem similar to the one solved by Grangeat [6] for planar projections in ℝ^3^. There are numerous approximate reconstruction methods, most of them using some back projection techniques (or numerical algorithms) and search for point sources as intersections of cone sheets reconstructed from coincidence measurements on the Compton camera. Lastly let us also cite some other original approaches based on statistical physics [36] as well as algebraic methods [37–39].

In this situation, it is of interest to find an analytic inversion of the *special* *𝒞*_2_-conical Radon transform responsible for the imaging process in Compton camera. Before tacking this problem, we discuss here a more general problem in which the vertex of the scattering cone is not constraint to lie on a plane (or a surface) as in the Compton camera case. This would give us some freedom to find an inversion formula for another class of *𝒞*_2_-conical Radon transform. We will come back to the Compton camera in another work. Thus, the mathematics of the Compton camera imaging process have led to a new class of conical Radon transform.

### 5.3. Properties of the *𝒞*_2_-conical Radon transform

**Figure 6:**
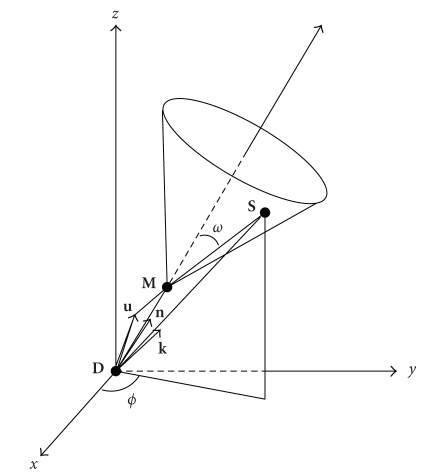
*𝒞*_2_-cone and *𝒞*_2_-conical Radon transform definition.

The *𝒞*_2_-cone is generated by the rotation of a straight line making an angle *ω* around an axis direction **n** and meeting this axis at vertex **M**; see Figure 6. Thus, **D** being the detection point, we have $\vec{DM}=M=pn$. Let **S** be a running point of the cone sheet and denote this point by $\vec{DS}=S=rk$. Let the angle *γ* be defined by (**k** ⋅ **n**) = cos *γ*. Thus, by considering trigonometric relations in the triangle *DMS*, one can write down the relation

(35) $$r=p\frac{\sin\omega }{\sin(\omega -\gamma )}=p\frac{\sin\omega }{\sin(\omega -\cos^{-1}(k\cdot n))},$$

which may be regarded as the cone equation in a meridian section within polar coordinates (*r*, *γ*) and polar axis **n**. At *ω* = *π*/2 we recover the equation of a plane as a degenerate cone [28].

When evaluating a *𝒞*_2_-conical projection, we integrate a nonnegative function *f* on one sheet of the cone. This is equivalent to saying that, giving a “mass” density, we compute the “mass” of a piece of cone surface limited by the intersection curve of the support of *f* with the cone. This “mass” may be calculated in any convenient coordinate system.

We will use the coordinate system which is expressed in (35). The area element of the cone is the product of the arc element of the line by the element of circle in a plane perpendicular to the cone axis.

The arc element is

(36) $$ds=\sqrt{{\left(dr\right)}^{2}+{\left(rd\gamma \right)}^{2}}=\frac{p\;\sin\;\omega }{\;\sin\;(\omega -\gamma )}\frac{d\gamma }{\;\sin\;(\omega -\gamma )}.$$

Now the element of circle is *r* sin *γ* *d* *ψ*. Hence, the cone area element is

(37) $$\begin{array}{cc} da & =r\;\sin\;\gamma d\psi \times \frac{p\sin\omega }{\;\sin\;(\omega -\gamma )}\frac{d\gamma }{\sin(\omega -\gamma )}\\ & =\;\sin\;\gamma d\psi \times {\left(\frac{p\;\sin\;\omega }{\;\sin\;(\omega -\gamma )}\right)}^{2}\frac{d\gamma }{\sin(\omega -\gamma )}. \end{array}$$

In the same coordinate system, a *𝒞*_2_-conical projection of *f* is expressed as the integral of *f* on the cone sheet with the integration measure *da*. *f* has then the expression *f* (*r*, Ω*_k_*), with Ω*_k_* = (*γ*,*ψ*), *r* = *p* sin *ω*/ sin (*ω* − *γ*) and angular ranges 0 < *γ* < *ω* and 0 < *ψ* < 2*π*. Thus,

(38) f^2(pn,ω)=∫f(psin⁡ ωsin⁡(ω−γ),Ωk)sin⁡ γdψ×(p sin⁡ ωsin⁡(ω−γ))2dγsin⁡(ω−γ),

or alternatively

(39) f^2(pn,ω)=∫(f(r,Ωk)r2)r=p(sin⁡ ω/sin⁡ (ω−γ))×sin⁡γdψdγsin⁡(ω−γ).

One may use the integration over a one-dimensional delta function to express the substitution of *r* by the cone equation (35):

(40) $$\int_{-\infty }^{\infty }dr\delta (r-p\frac{\sin\;\omega }{\sin(\omega -\gamma )}).$$

Since

(41) $$\delta (r-p\frac{\sin\;\omega }{\sin\;(\omega -\gamma )})\frac{1}{\sin(\omega -\gamma )}=\delta (p\;\sin\;\omega -r\;\sin\;(\omega -\gamma )),$$

and *r* ^2^ *d* *r* sin *γ* *d* *ψ* *d* *γ* = *r* ^2^ *d* *r* *d*Ω*_k_* =*d* **S**, we can replace the original integration range:

(42) {r∈ℝ, 0<γ<ω, 0<ψ<2π},

by

(43) $$\begin{array}{cc} \{ & r\in {\mathbb{R}}_{+},\;\Gamma =[0<\gamma <\omega \cup \pi <\gamma <\omega +\pi ],\\ & S^{1}=[0<\psi <2\pi ]\}, \end{array}$$

which fits in the chosen spherical coordinate system with **n** as polar axis.

The *𝒞* ^2^-conical projection of *f* has now the Fredholm form of the first kind with a delta function kernel. In intrinsic vector notations, it reads

(44) $${\hat{f}}_{2}(pn,\omega )=\int dSf(S)K_{2}(pn,\omega |S),$$

where

(45) $$K_{2}(pn,\omega |S)=\delta (p\;\sin\;\omega -r\;\sin\;(\omega -\cos^{-1}(k\cdot n))).$$

(Compare with the previous case, see (18), (19)).

Finally, at *ω* = *π*/2 we recover precisely the planar Radon projection in ℝ^3^:

(46) $${\hat{f}}_{2}(pn,\frac{\pi }{2})=\int dSf(S)\delta (p-r(k\cdot n)).$$

### 5.4. A central slice-like theorem

Again as in Section 4, we may use the Fourier decomposition of the delta function^1^

(47) $$\begin{array}{cc} \delta (p\;\sin\;\omega -r\;\sin(\omega -\gamma )) & =\int_{-\infty }^{\infty }d\nu \;\exp-2i\pi \nu (p\;\sin\;\omega -r\;\sin\;(\omega -\gamma )), \end{array}$$

together with the decomposition of *f* into spherical components

(48) $$f(x,y,z)=f(r,\Omega_{k})=\sum_{l,m}f_{lm}(r)Y_{lm}(\Omega_{k}),$$

where, in the chosen spherical coordinate system, Ω*_k_* denotes the direction parameters of the unit vector **K** Hence, inserting (47) and (48) in (44) we obtain

(49) f(pn,ω)^=∑l,m∫−∞∞dνe−2iπν(psin⁡ω) ×∫0∞r2drflm(r)∫Γ∪S1dΩkYlm(Ωk)e2iπνrsin⁡(ω−γ).

Recall cos *γ* = (**k** ⋅ **n**). As sin (*ω* − *γ*) = cos (*π*/2 − *ω* + *γ*), we introduce the decomposition of a plane wave in space in spherical components (see [40], page 471):

(50) $$\begin{array}{cc} e^{2i\pi \nu r\cos(\pi /2-\omega +\gamma )} & =\underset{n}{\sum }i^{n}(2n+1)j_{n}(2\pi \nu r)P_{n}(\cos(\frac{\pi }{2}-\omega +\gamma )), \end{array}$$

where *j_n_* (*x*) is the spherical Bessel function of order *n*.^2^

Let **u** be a fixed unit vector, defined by Ω_u_, such that cos (*π*/2 − *ω* + *γ*) = (**k** ⋅ **u**). Then the Legendre polynomial *P*_n_ ( cos (*π*/2 − *ω* + *γ*)) can be further expanded in terms of spherical harmonics [25]:

(51) $$\begin{array}{cc} P_{n}\cos(\cos(\frac{\pi }{2}-\omega +\gamma )) & =\frac{4\pi }{2n+1}\overset{m^{\prime }=n}{\underset{m^{\prime }=-n}{\sum }}Y_{nm^{\prime }}^{*}(\Omega_{k})Y_{nm^{\prime }}(\Omega_{u}). \end{array}$$

Putting relation (51) in (50) and integrating on *d*Ω _*k*_, since the range of *ϕ* is 𝕊^1^, we can write

(52) $$\int_{\Gamma \cup S^{1}}d\Omega_{k}Y_{lm}(\Omega_{k})Y_{nm^{\prime }}^{*}(\Omega_{k})=\delta_{mm^{\prime }}K_{ln}^{m}(\omega ),$$

with |*m*| < inf(*l*,*n*). Thus, the result follows:

(53) f(pn,ω)^=∑n,mYnm(Ωu)∫−∞∞dνe−2iπν(psin⁡ω)4πil∫0∞r2dr ×∑ljl(2πνr)Klnm(ω)flm(r).

Right-hand side of (53) expresses $\hat{f(pn,\omega )}$ as a spherical component expansion of the *𝒞* ^2^-conical Radon transform in terms of the variable *p* sin *ω* **u**, instead of *p* **n**. So if the *𝒞* ^2^-conical data can be rewritten under the form of a function of *p* sin *ω* **n** and of *ω*, with a spherical component decomposition, that is,

(54) $$\hat{f(pn,\omega )}=\hat{g(p\;\sin\;\omega u,\omega )}=\sum_{lm}\hat{g_{lm}(p\;\sin\;\omega },\omega )Y_{lm}(\Omega_{u}),$$

then we can extract the new data spherical component as

(55) glm(psin⁡ω^,ω)=∫−∞∞dνe2iπν(psin⁡ω)4πil∫0∞r2dr ×∑ljl(2πνr)Klnm(ω)flm(r).

Now by Fourier inverting with respect to *q* = *p* sin *ω* the two sides of (55), (since sin *ω* > 0 for 0 < *ω* < *π*), we find that

(56) sin⁡ω∫−∞∞dqglm(q^,ω)e2iπνq=4πil∫0∞r2dr∑ljl(2πνr)Klnm(ω)flm(r).

Next, by appropriately choosing **u**, a generalized Hankel identity may be derived. Recall that for spherical Bessel functions (see, e.g., [25]) this identity is of the form

(57) $$\frac{1}{k^{2}}\delta (k-k^{\prime })=\int_{0}^{\infty }\rho^{2}d\rho 4\pi j_{l}(2\pi k\rho )4\pi j_{l}(2\pi k^{\prime }\rho ).$$

This new identity allows to extract the spherical component *f_l__m_*(*r*) of *f* and thereby achieve inversion (details will be presented elsewhere). Equation (54) relates implicitly *p* and *ω* and may suggest a new type of gamma camera realization, which should be investigated. Finally for *ω* = *π*/2, (44) shows that this ${𝒞}_{2}$-conical Radon transform is just the Radon transform in ℝ^3^. The whole procedure goes through with drastic simplifications since the natural choice for **u** is **n**, thus $\hat{f(pn,\omega )}=\hat{g(pu,\omega )}$.

## 6. CONCLUSIONS AND PERSPECTIVES

The Radon transform has enjoyed tremendous popularity in imaging science as it has been extended, generalized in pure mathematics (see, e.g., [19, 41, 42]) as well as exploited in many fields of applications [17, 43, 44]. In this paper we have presented two further generalizations of the Radon transform, namely, two classes of conical Radon transforms which originate from imaging processes using Compton scattered radiation. The first class, called *𝒞*_1_-conical Radon transform, is related to an imaging principle with a collimated gamma camera whereas the second class, called *𝒞*_2_-conical Radon transform, contains a special subclass which models the Compton camera imaging process. We have also shown that inversion of *𝒞*_2_-conical Radon transform can be achieved under a special condition which is not yet, for the moment, implemented in gamma-ray emission imaging science. Exploiting scattered radiation to reinforce sensitivity as well as enlarging field of view and cutting down operating time may lead to employing large scattering medium but without collimator for gamma cameras. The mathematics behind this perspective will be based on a yet little known Radon transform: the torus Radon transform, which may bring even more exciting mathematical topics to be explored in the years to come.

## Footnotes

^1^As the delta function is an even function, the sign of the exponential under the integration sign may be chosen at will.

^2^This is valid for *ν* > 0, otherwise we take the Fourier representation of the delta function with a different sign in (47) as indicated in the previous footnote.
